# An online randomised controlled trial to evaluate the effectiveness of an online intervention for parents/guardians of children aged 4–7 years old who are concerned about their children’s emotional and behavioural development: EMERGENT study

**DOI:** 10.3389/fpsyg.2026.1819302

**Published:** 2026-06-19

**Authors:** Jowinn Chew, Daniel Frings, Paula Reavey, Clare Allabyrne, Claudiu Herteliu, Chris Flood, Lucy Watson

**Affiliations:** 1 London South Bank University, London, United Kingdom; 2 Bucharest University of Economic Studies, Bucharest, Romania

**Keywords:** children, digital health, digital mental health interventions, mental well-being, parental intervention, randomised controlled trials

## Abstract

**Background:**

Many young children experience emotional and behavioural difficulties, yet access to early support is restricted by long waiting times and financial and logistical barriers. Digital mental health interventions (DMHIs) offer scalable support, but few are designed to engage both parents and children collaboratively. Embers the Dragon is a self-guided parent–child programme designed to help support the emotional wellbeing of parents/guardians and children aged 4–7. Aims: This study evaluated the effectiveness, acceptability, and health economic impact of Embers the Dragon compared with treatment as usual (TAU).

**Methods:**

A two arm online randomised controlled trial allocated 456 parents/guardians to Embers (*n* = 235) or control (*n* = 221). Assessments were completed at baseline, 8, 16, and 24 weeks. Primary outcomes were the Strengths and Difficulties Questionnaire (SDQ) and Parents Sense of Competence (PSOC). Secondary outcomes included the Parenting Scale (measuring parental discipline) and EuroQol Five Dimensions of health (EQ-5D-3L). Analyses followed an intention to treat mixed effects model.

**Results:**

Participants in the Embers condition reported significant reductions in SDQ scores from baseline to 24 weeks; the control group showed no improvement. Parental confidence increased in both conditions, with greater improvements shown in the Embers condition at 16 weeks, although differences were not maintained at 24 weeks. Parenting discipline improved across conditions, with more pronounced improvements for the Embers condition by 24 weeks. EQ5D3L scores showed no meaningful change over time and did not differ between conditions. Health economic analysis indicated that Embers was less costly and more effective than TAU, producing net savings per unit improvement in both SDQ and self-efficacy outcomes.

**Conclusion:**

Embers the Dragon delivered meaningful improvements in children’s emotional wellbeing, as well as parenting confidence and discipline responses. As a low intensity, self-guided programme, it shows promise as a scalable early intervention option.

**Clinical trial registration:**

https://osf.io/ybxzu/ and ISRCTN (ISRCTN58327872).

## 1 Introduction

Childhood mental health issues represent a significant and escalating public health concern. In the United Kingdom (UK), 1 in 5 children and young people experience emotional or behavioural difficulties that interfere with daily functioning (Benton et al., 2021; Children’s Commissioner, 2021; Clark et al., 2022; Domhardt et al., 2021; Waite et al., 2021). These difficulties can emerge as early as 2 years of age and, unaddressed, can persist and evolve into more complex problems later in life with serious and lasting consequences (Butler et al., 2022; Colizzi et al., 2020; Jeong et al., 2021; Lehtimaki et al., 2021; McGorry and Mei, 2018). Long-term outcomes include an increased risk of developing mental health disorders in adulthood (Bitsko et al., 2022; Copeland et al., 2009, 2014, 2021; Mulraney et al., 2021; Otto et al., 2021), substance use (Goodman, 2010; Groenman et al., 2017; Mason et al., 2004), violent behaviours (Mason et al., 2004), and reduced quality of life (Butler et al., 2022; Copeland et al., 2014; Kieling et al., 2024).

These difficulties have far-reaching implications. Children with probable mental health disorders are three times more likely to miss over 15 days of school annually compared to peers, with mental ill health now recognised as a leading cause of authorised absences and educational disengagement (Children’s Commissioner, 2022; Office for National Statistics, 2025). At the same time, demand for mental health services has exceeded available capacity, placing significant strain on existing systems, with over 270,000 children waiting for support and thousands facing delays of over 2 years (Children’s Commissioner, 2024a). These challenges are compounded by regional inequalities and underinvestment, with average spending on children’s mental health services remaining disproportionately low (Children’s Commissioner, 2025). Without timely intervention, these issues contribute to long-term economic costs through increased healthcare utilisation, reduced workforce participation, and greater reliance on welfare systems (Liverpool et al., 2025; Pollard et al., 2023).

Early intervention is widely recognised as critical to improving children’s long-term mental health outcomes (Butler et al., 2022; World Health Organization, 2020). Despite this, access to timely and effective care remains limited, and the COVID-19 pandemic has further exacerbated the strain on already overstretched services (Newman et al., 2024; Ng and Ng, 2022; Reed et al., 2023). A 2024 report by the UK’s Children’s Commissioner revealed that nearly 50,000 children and adolescents in the United Kingdom are referred to National Health Service (NHS) mental health services each month. Of these, over a quarter are still waiting for support, and almost 40% have their referrals closed without receiving help (Children’s Commissioner, 2024b; Crenna-Jennings and Hutchinson, 2020; Khunti et al., 2023). Families are thus often left to navigate complex and under-resourced systems with minimal guidance, contributing to heightened parental stress and poorer mental health outcomes for children (Ghafari et al., 2022; Koet et al., 2022; Koning et al., 2019; McGorry and Mei, 2018; O’Brien et al., 2016; Sayal and Taylor, 2004; Selby et al., 2021; Sourander et al., 2001). This strain also has significant repercussions for the mental health of parents/guardians themselves, with elevated levels of anxiety (Martin et al., 2024) and stress (Meng and Wiznitzer, 2024) commonly reported among caregivers of children with mental health difficulties.

Digital mental health interventions (DMHIs) have emerged as a potential solution to address the gap in children’s mental health service availability (Flujas-Contreras et al., 2019; Khanna and Carper, 2022; Liverpool et al., 2020; Wies et al., 2021). While not intended to replace face-to-face services, DMHIs potentially offer scalable, accessible support for families who may not require intensive intervention (Gardiner, 2025; Graham et al., 2020; Schueller and Torous, 2020). DMHIs provide several advantages, including scalability, ease of access, and the ability to deliver support to families who may be underserved by traditional interventions due to geography, cost, stigma, or time constraints (Lehtimaki et al., 2021; Peskin et al., 2024; Piers et al., 2023) without the need for therapist time, formal support structures, or in-person interventions (Graham et al., 2020; Peskin et al., 2024).

Despite the growing potential of digital mental health interventions (DMHIs), most existing tools focus exclusively on either the child or the parent, potentially overlooking the advantages of integrated approaches that engage both parties (Chew et al., 2026; Dittman et al., 2014; Jabbari et al., 2024; McAloon and Armstrong, 2024; Satyanarayana et al., 2024; Thongseiratch et al., 2020). Collaborative tools that involve both children and parents not only foster engagement and shared responsibility but also increase the likelihood that intervention strategies are embedded into daily routines and sustained over time (Barmish and Kendall, 2005; Chew et al., 2026; Csirmaz et al., 2024). While parent–child interventions do exist, they are typically delivered face-to-face or require therapist support, which limits their scalability and accessibility (Brendel and Maynard, 2014; Csirmaz et al., 2024; Dowell and Ogles, 2010; Fisak et al., 2023; Helps and Le Coyte Grinney, 2021; Lo et al., 2023; March et al., 2009; Shortt et al., 2001; Spence et al., 2006). This gap is reflected in a recent systematic review which screened over 3,703 unique records evaluating DMHIs for children and families and identified only three studies that targeted both children and adults through combined digital components (Chew et al., 2026). While all three were randomised controlled trials, two were assessed as having a high risk of bias, underscoring the urgent need for more rigorous and inclusive research in this area.

There is a clear need for scalable, evidence-based digital programs that support collaborative learning between parents and children in the home environment. Addressing this lacuna is important as it will inform the development of effective early intervention DMHIs targeting children’s emotional wellbeing, designed to be delivered without the need for therapists’ time. Such interventions have the potential to alleviate the burden on overstretched services while providing accessible support to families facing barriers to existing care pathways. Moreover, effective interventions that target both children and their families may help prevent the escalation of mild to moderate emotional and behavioural difficulties into more severe problems, with benefits extending to broader systems such as schools and communities. The current study evaluates the efficacy of one such intervention.

To address the need for effective, scalable, and collaborative DMHIs, the current trial aims to evaluate one such product: “Embers the Dragon”. This is a self-guided digital program designed to support the emotional wellbeing of parents/guardians and children aged 4–7 through a collaborative approach. The program is grounded in social learning theory (Bandura, 1977), which is a well-established and extensively applied theoretical framework for early childhood and parenting interventions (Jeong et al., 2021). Embers the Dragon uses joint parent–child activities that focus on modelling and reinforcement. These activities aim to help parents support the development of social–emotional skills in their children. A central feature of the program is a series of animated stories. These follow Embers, a cartoon dragon, and his friends as they navigate common childhood challenges. The stories are paired with psychoeducational videos and resources for parents. These cover key topics such as child development, modelling, reinforcement, and behaviour management strategies. The results of a feasibility study trialling Embers the Dragon program revealed that 98% of parents/guardians reported a significant improvement in their self-assessed parental effectiveness and confidence in responding to their children’s emotional needs (Selby et al., 2021). Qualitative feedback also highlighted that children were able to successfully recall coping strategies highlighted in the program (Selby et al., 2021).

Building on the above findings, the current randomised controlled trial aims to evaluate the accessibility, efficacy, and acceptability of Embers the Dragon in real-world settings. This will provide much needed RCT level evidence around the efficacy of digital interventions which target both parents and children, while also speaking to the efficacy of the specific product. It was hypothesised that at 24 weeks post randomisation: (1) participants’ parenting sense of parenting efficacy (measured via the Parents Sense of Competence, Johnston and Marsh, 1989) will be higher in the Embers condition compared to the control and (2) the level of children’s social, emotional and behavioural difficulties will be lower in the Embers condition compared to the control measured via the Strengths and Difficulties questionnaire (Goodman, 2011). As a secondary outcome, we also tested the impacts of the intervention on parental discipline styles (via the Parenting Scale, Arnold et al., 1993), hypothesising increased reduction in lower laxness, over reactivity and verbosity in the Embers condition over time, relative to control.

This trial and its data analysis plan were pre-registered on the Open Science Framework^1^ and via protocol publication (Frings et al., 2024). Alongside testing the hypothesis above, we also conducted a pre-registered health economic analysis,^2^ which aimed to examine the health economic impact of Embers compared to Treatment as Usual (TAU). Data files and syntax are available at https://osf.io/4xjt9.

## 2 Methods

### 2.1 Ethical approvals

The study received ethical approval from the London South Bank University Ethics Panel (ETH2324-0004) and the Central Bristol Research Ethics Committee (24/SW/0003).

### 2.2 Study design

The current study was originally designed and pre-registered as a three-armed, intention to treat, randomised control trial comprising: (i) a control condition, (ii) a home-based Embers condition in which participants would access the Embers program in a parent-led setting, and (iii) a school-based Embers condition in which teachers would deliver the Embers program in classroom settings, fulfilling personal, social, health and economic (PSHE) education requirements (Department of Education, 2019). This design would have allowed for comparisons between the school-based and home-based conditions to isolate the impact of the school delivery context, and between the school-based and control conditions to assess overall efficacy relative to treatment as usual. However, despite several attempts to contact approximately 1,836 schools across the UK, only nine schools agreed to participate. From these, only 36 parents enrolled on the trial. This limited uptake rendered the school-based condition insufficiently powered for robust analysis, and it was therefore excluded from the final trial design. As a result, the analysis proceeded with only two conditions: the control, and the home-based Embers condition (referred to as the Embers condition). Assessments were conducted at baseline, 8-weeks, 16-weeks, and 24-weeks follow up. 24 weeks was the primary endpoint, with secondary endpoints at 8 and 16 weeks. Statistical power was maintained by recruiting an increased number of participants into the remaining two arms, such that we were powered to detect expected effect sizes (see 2.9 below for full details). Qualitative interviews were also conducted to support a robust mixed methods process evaluation. Finally, a health-economic evaluation was conducted to assess the cost-effectiveness of the Embers intervention by capturing service use, indirect costs, delivery costs, downstream savings, and quality-of-life- outcomes.

To ensure methodological rigour and transparency, alongside protocol publication (Frings et al., 2024), the trial and data analysis plan were pre-registered on both ISRCTN (ISRCTN58327872) and the Open Science Framework.^3^ Data and other outputs can be found on the projects main OSF site.^4^

### 2.3 Inclusion and exclusion criteria

All participants were screened against the following criteria:

#### 2.3.1 Inclusion criteria

1. Parents/guardians of children aged 4–7 who are concerned about their children’s mental/emotional well-being, including both those who are and are not already actively seeking professional support.
2. Both parent and child fluent in English.
3. Access to a program-compatible digital device.
4. Willingness to complete follow-up measures.

#### 2.3.2 Exclusion criteria

1. Previous experience with the Embers the Dragon program.
2. Currently undergoing a treatment intervention with CAMHS or social care.
3. Shares parenting/caring duties for the same child for which a parent/guardian is already recruited.
4. Already recruited to the study in relation to a different child.
5. Previous involvement in Patient and Public Involvement and Engagement (PPIE) work associated with Embers.

### 2.4 Randomisation

Participants were randomised to either the Embers or control condition stratifying by age and gender of the child using block randomisation. To achieve this, the *blockrand* command from the blockrand R package was used. The block size was randomised between 1 and 4, and 68 slots per stratification permutation generated. The randomisation ratio is 1:1 between the conditions. Given that baseline data were collected as part of the app usual operation (i.e., the measures were used as part of the standard onboarding), randomisation occurred prior to baseline data being collected.

### 2.5 The embers the dragon program

The Embers the Dragon program is a psychoeducation digital program developed to support parents/guardians, children aged 4–7 and schools in developing emotional well-being in early years. As noted above, the program is based on social learning theory and uses activities focusing on modelling and reinforcement to encourage parent-supported development of social–emotional skills in children. The program consists of 12 episodes, each 6 to 7 min long approximately. Each episode is comprised of a story following the adventures of Embers the Dragon and his friends as they explore common challenges of childhood. Parents/Guardians are asked to watch each episode with their child. To accompany the episodes, there are also resources such as parenting advice videos, worksheets, and games which parents/guardians can review with their child to help them develop their behavioural and communication skills. The school version of the Embers the Dragon program (not tested here, see *Study Design*, above) includes all the same features, along with lesson plans and classroom resources that can be adapted for a whole class, small group, or one-on-one settings.

### 2.6 Primary outcomes

#### 2.6.1 Parental efficacy

To test the impact of Embers on parents’ sense of parenting efficacy, the Parents Sense of Competence (PSOC) was used. The PSOC aims to measure parent’s perceived confidence in their parental skill in supporting their children. The measure comprises five items rated on a 5-point Likert scale. The items are summed to yield a total score, with higher scores indicating higher levels of parental self-efficacy (Johnston and Marsh, 1989). Cronbach’s *α* = 0.80 in the current study.

#### 2.6.2 Children’s social, emotional and behavioural difficulties

To test impacts on children receiving the intervention, the Strengths and Difficulties questionnaire (SDQ) (Goodman, 2011) was used. This paper presents data based on a modified time reference, using 1 month instead of 6 months reference.^5^

The SDQ aims to assess the behaviours, emotions and relationships of children and young people (aged 4–17 years). The scale comprised 25 items, divided between five scales (emotional symptoms, conduct problems, hyperactivity/inattention, peer relationship problems and prosocial behaviour). Parents were asked to rate each item based on their child’s behaviour over the past month, selecting “Not True,” “Somewhat True,” or “Certainly True. In line with our pre-registration, and to avoid increased Type 1 error risk, we analysed total scale scores. Lower scores on the emotional symptoms, conduct problems, hyperactivity/inattention and peer relationship problem scales indicate a lower level of difficulty, whereas scoring is reversed for the prosocial behaviour scale, with higher scores indicating lower levels of difficulty (Goodman, 1997). Cronbach’s *α* = 0.76 in the current study.

#### 2.6.3 Qualitative interviews

Interviews with parents/guardians and children were conducted face to face or online to explore the lived experience of the intervention. These interviews use a visual-qualitative technique involving images and video excerpts from Embers relating to key characters and storylines. The visual element is part of an elicitation technique to help prompt and facilitate thought and memory recall (Reavey, 2020). Anonymised transcripts from these interviews can be found at https://osf.io/xzaj7. Findings from the subsequent analysis will be published elsewhere (and the outputs also lodged on the project OSF site).

#### 2.6.4 Health economic analysis

The overall aims of our health economic evaluation package were to (i) estimate the monetary cost per unit of identified health outcome improvement, (ii) examine wider cost implications (i.e., savings made elsewhere, such as fewer GP appointments), and (iii) provide comparative data in a way that can be examined in relation to the cost effectiveness of other interventions. Specifically, we will measure occurrences of self-reported GP visits, school family liaison and social service appointments by arm, including estimates of mental health-related indirect/out of pocket expenses incurred by families.

### 2.7 Secondary outcomes

#### 2.7.1 Parental discipline

The Parenting Scale aims to assess parental discipline responses over the last 2 months. The measure comprised 30 items rated on a 7-point Likert scale. These items are divided into three subscales (laxness, over-reactivity and verbosity). As above, total scale scores were analysed. Lower scores indicate good parental responses, and high scores indicate dysfunctional parental responses (Arnold, O’Leary, Wolff and Acker, 1993). Cronbach’s α for the whole scale = 0.83 in the current study.

#### 2.7.2 Health questionnaire (EQ-5D-3L)

To support health economic analysis, the EQ-5D-3L is a widely used survey which assesses health-related quality of life. The measure comprised six subscales (mobility, self-care, usual activities, pain, discomfort and anxiety/depression). Individuals rate their level of problems for each scale (no problems, moderate problems and severe problems). Additionally, the measure includes a visual analogue scale where the highest endpoint is labelled as ‘The best health you can imagine’ (100 points) and the lowest as ‘The worst health you can imagine’ (0 point) (Herdman et al., 2011). Cronbach’s α = 0.81 in the current study. While the measure was primarily included to support health economic modelling, full outcome analyses were conducted in line with pre-registration and standard RCT reporting practices to ensure transparency and allow for exploratory interpretation.

#### 2.7.3 Health impact survey

To support the health economics analysis, participants were asked to indicate what impact their child’s mental health has had on various aspects of the parents/guardians’ (and their families) lives. The impact survey comprised three questions: (1) how many attendances to the GP, family school liaison and social services have you made resulting from concerns over your child’s mental health? (2) details of any other interventions used (if any)? and (3) an estimate of any additional financial cost associated with managing your child’s mental health (e.g., including loss of time at work, appointment travel or additional care)?

#### 2.7.4 Needs and hopes questionnaire

Participants were be asked to indicate what led them to enrol in the study and what they hope to achieve by using the Embers platform. The Needs and Hopes Questionnaire comprised three questions: (1) what do you think are your child’s main needs at the moment which you might want to focus on supporting, (2) what do you hope will be different after using the Embers platform and (3) what areas do you most want to work on? This information will be used to explore the lived experience of the intervention. Participants in the control condition were only administered the first question, while participants in the Embers condition were administered all three questions.

#### 2.7.5 Parental engagement

Parental engagement was defined as the frequency and duration of shared activities between parents and children. This was measured via self-reported hours per week they spend playing, reading, and sharing meals with their child.

#### 2.7.6 Platform engagement

Participant engagement with the Embers platform was assessed using multiple metrics including the number of logins, duration of each session, number of episodes viewed, and exercises accessed.

### 2.8 Procedure

Participants were recruited via several channels. We undertook promotion of the study on social media (paid Facebook and Instagram adverts, also organic posts). NHS GPs (family doctors) also sent a short message promoting their study to all parents with children in eligible age ranges. Finally, interested community sites (children’s centres, etc) displayed posters advertising the study. All participants provided informed consent for the collection and analysis of their data. Participants were heard about the study through the following sources: Social media (43.6%), General Practitioners (GPs) (36.1%), word of mouth (12.4%), their child’s school (3.2%, independent of the school arm condition), and other non-specified sources (4.7%). If eligible, participants were automatically redirected to the study information sheet and consent form. Upon providing informed consent, participants were randomly assigned to either the Embers or control condition. Those in the Embers condition received an email with instructions for downloading the Embers program and were prompted to complete the baseline survey after installation (via the app). Participants in the control condition received an email with a link to the baseline survey (via Qualtrics). Text and presentation were identical between modes.

All participants received three email/app reminders to complete the baseline and each follow-up survey at each stage (reminder number, presentation and wording were matched, with only delivery mode differing). Additionally, a text message reminder was sent 2 weeks before the due date of their final (24-week) follow-up survey. Participants in the control condition were given access to the Embers program at the end of the study. Participants received £10 for each completed survey (baseline, 8, 16, and 24 weeks), with an additional £20 incentive for completing all surveys. Payments were issued via Tango vouchers (see tangocard.com).

### 2.9 Sample size

Pilot feasibility work (Selby et al., 2021) revealed an effect size of *d* = 0.51 (a medium effect size, equivalent to effect size *f* = 0.26) between pre and post measures in the Embers condition in parental confidence, and no difference over time in the control. Our planned smallest comparative sample of *n* = 213 was sufficient to detect an interaction effect of *f* = 0.10 (assuming 2 groups, two measurement points, power = 0.80). It is sufficient to detects a difference between groups at effect size *d* = 0.37 (assuming power = 0.80, two tailed tests, 1:1 ratio of completed cases).

### 2.10 Data analysis approach

The analyses were conducted on an intention-to-treat basis, including all participants who provided baseline data, as randomised. Separate analyses were conducted for each outcome. Differences between conditions were tested using a mixed effects model, with Intervention (Embers vs. Control), Time (Baseline vs. 24 Weeks), and their interaction included as fixed effects. Individual-level variation was accounted for by including participants as a random effect. The primary contrast of interest was the interaction between intervention, condition, and time, specifically comparing baseline to 24-week outcomes. The mixed effects model approach makes no assumptions around patterns of missing data (i.e., it does not assume data are missing at random).

Health economic analyses were conducted using data from the participant survey. These were combined with direct delivery cost information, plus estimates of downstream savings related to the outcomes benefits realised. Data was collected from the same sample who undertook for a detailed description of the analyses.

## 3 Results

### 3.1 Participant flow

**Figure 1:**
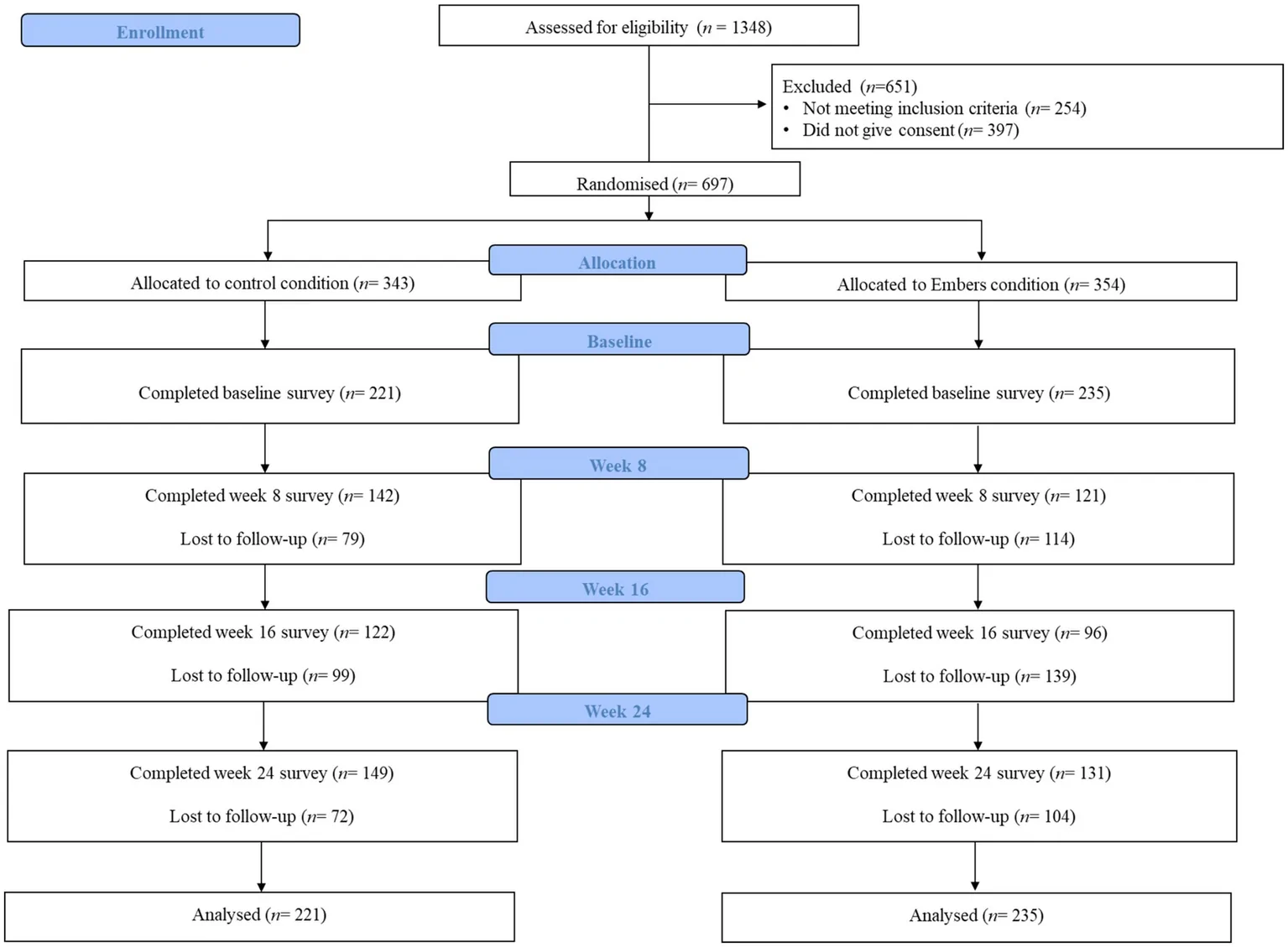
CONSORT flow diagram.

Participant flow through the study can be seen in Figure 1.

### 3.2 Data preparation quality assurance

Data processing (collapsing multiple datasets, transposing data between columns and rows, etc) was required to undertake the analysis planned. To ensure accuracy, 10% of the post transformation data (*n* = 73) were manually cross-checked against the raw data. Four items per participant were checked. No discrepancies were identified, confirming the accuracy of the data transformation process.

### 3.3 Participant demographics

Table 1 presents the demographic characteristics of the participants. A total of 456 participants were recruited at baseline, with 235 randomly allocated to the Embers condition, and 221 to the control condition. It is important to note a small number of participants did not respond to every demographic question. The number of respondents varied by item as follows: parent age (*n* = 448), parent gender (*n* = 456), ethnicity (*n* = 455), sexual orientation (*n* = 455), relationship status (*n* = 455), living situation (*n* = 455), family role (*n* = 455), child age (*n* = 438), child gender (*n* = 438), child school year (*n* = 455), child disability status (*n* = 456), and child special educational needs (SENS) (*n* = 456). The majority of parents/guardians were female (96.1%), white (85.3%), heterosexual (91.9%), and married (63.5%). Their average age was 39.9 years (SD = 5.41). Most parents/guardians (97.1%) reported that the child enrolled in the study lived with them full-time, and the majority identified as the child’s mother (95.8%). The average age of the children enrolled in the study was 5.5 years (SD = 1.1). The majority of children were male (56.6%), in Year 1 (28.1%), and were reported to have no disability (77.6%) or special educational needs (75.4%). Participants were evenly distributed across socioeconomic quintiles, with an average Index of Multiple Deprivation (IMD) decile of 5.7 (SD = 2.7), indicating a varied combination of deprivation levels.

**Table 1:** Participant characteristics at baseline.

Variable	Control (*n* = 221)	Embers (*n* = 235)
Age, M ± SD (range)	40.0 ± 5.5 (27–61)	39.8 ± 5.3 (25–58)
Gender, *n* (%)		
Female	212 (95.9)	226 (96.2)
Male	8 (3.6)	9 (3.8)
Non-binary/third gender	1 (0.5)	0
Ethnicity, *n* (%)		
White	193 (87.3)	195 (83.0)
Mixed/Multiple ethnic groups	3 (1.4)	12 (5.1)
Asian/Asian British	11 (5.0)	13 (5.5)
Black/African/Caribbean/Black British	7 (3.2)	6 (2.6)
Other ethnic group	5 (2.3)	6 (2.6)
Prefer not to say	1 (0.5)	3 (1.3)
Sexual orientation, *n* (%)		
Straight	206 (93.2)	212 (90.2)
Bisexual	6 (2.7)	11 (4.7)
Gay	2 (0.9)	4 (1.7)
Other	2 (0.9)	2 (0.9)
Prefer not to say	4 (1.8)	6 (2.6)
Relationship status, *n* (%)		
Married	130 (58.8)	159 (67.7)
In a relationship	45 (20.4)	43 (18.3)
Single	29 (13.1)	23 (9.8)
Divorced	7 (3.2)	1 (0.4)
Separated	5 (2.3)	6 (2.6)
Other	2 (0.9)	1 (0.4)
Prefer not to say	2 (0.9)	2 (0.9)
Living situation, *n* (%)		
The child enrolled in the study lives with me all the time	211 (95.5)	231 (98.3)
The child enrolled in the study lives with me some of the time	8 (3.6)	3 (1.3)
Other	1 (0.5)	1 (0.4)
Family role, *n* (%)		
Mother	209 (94.6)	227 (96.6)
Father	6	8 (3.4)
Grandparent	2 (0.9)	0
Foster parent	1 (0.5)	0
Other	1 (0.5)	0
Prefer not to say	1 (0.5)	0
Child age M ± SD (range)	5.5 ± 1.1 (4–7)	5.5 ± 1.1 (4–7)
Child gender		
Female	96 (43.4)	92 (39.1)
Male	125 (56.6)	123 (52.3)
Child school year, *n* (%)		
Nursery/pre-school	18 (8.1)	11 (4.7)
Reception	51 (23.1)	56 (23.8)
Year 1	56 (25.3)	72 (30.6)
Year 2	52 (23.5)	56 (23.8)
Year 3	37 (16.7)	39 (16.6)
Year 4	2 (0.9)	0
Home-school/Other	4 (1.8)	1 (0.4)
Child disability		
Yes	48 (21.7)	54 (23.0)
No	173 (78.3)	181 (77.0)
Child Special Educational Needs (SENS)		
Yes	51 (23.1)	61 (26.0)
No	170 (76.9)	174 (74.0)
Socioeconomic Status (SES) by Index of Multiple Deprivation (IMD) Quintile (%)		
Q1: Deciles 1–2 (most deprived)	14.9	14.0
Q2: Deciles 3–4	21.7	22.6
Q3: Deciles 5–6	21.3	23.8
Q4: Deciles 7–8	21.3	18.3
Q5: Deciles 9–10 (least deprived)	20.8	21.3

### 3.4 Retention rates

The retention rates for the Embers and control conditions were 58.4% (n = 220) and 62.1% (n = 215) at baseline, 51.5% (*n* = 121) and 64.5% (*n* = 142) at week 8, 40.9% (*n* = 96) and 55.5% (*n* = 122) at week 16, and 55.3% (*n* = 130) and 67.3% (*n* = 148) at week 24, respectively. See Figure 1 for CONSORT diagram indicating full participant flow details.

### 3.5 Withdrawals

Seven participants withdrew from the study. Of these, two cited being too busy to continue, three reported that their child did not like the app or a specific feature of it, and the remaining two did not provide a reason. During the study, 16 potential adverse events were reported. Each was recorded in a adverse events log, and the research team contacted the participant to assess if the event was related to taking part in the study and to signpost them to relevant mental health services. All participants confirmed that none of the reported events were related to study participation.

### 3.6 Randomisation check

Independent samples *t*-tests were conducted to examine baseline differences between the Embers and control conditions on child age and child gender. The results indicated no significant differences in child age, *t* (423) = 0.27, *p* = 0.787, Cohen’s *d* = 0.03, or child gender, *t* (423) = 0.39, *p* = 0.694, Cohen *d* = 0.04 suggesting that randomisation was successful we also undertook randomisation analysis on the primary and secondary outcomes (see Table 2). The SDQ differed at baseline [*t* (428) = 2.58 *p* < 0.010, Cohen’s *d* = −0.25], with lower SDQ scores in the control condition than the intervention. No significant differences were observed in PSOC, [*t* (441) = 0.79, *p* = 0.430, Cohen’s *d* = 0.075]. There were differences in Parental Discipline scores, [*t* (435) = 2.08, *p* = 0.039, Cohen’s *d* = −0.20]. As we used participant as a random effect in our planned analysis (which controls for any baseline differences), no further adjustments were made to account for the differences in SDQ scores.

**Table 2:** Means, standard deviations, and 95% CI for all outcome measures.

Outcome measures	Embers Mean (SD), 95% CI	Control Mean (SD), 95% CI
PSOC		
W0	19.30 (3.16), 18.87–19.73	19.71 (2.76), 19.33–20.08
W8	19.86 (2.96), 19.30–20.42	20.01 (2.78), 19.54–20.47
W16	20.64 (2.73), 20.06–21.23	19.92 (2.95), 19.39–20.46
W24	20.45 (3.25), 19.86–21.05	20.24 (2.95), 19.76–20.72
SDQ		
W0	17.21 (4.02), 16.66–17.76	15.61 (6.20), 14.73–16.48
W8	16.75 (3.67), 16.06–17.45	15.20 (6.50), 14.09–16.32
W16	16.87 (4.08), 15.99–17.75	15.68 (6.88), 14.41–16.96
W24	15.99 (5.52), 14.97–17.01	16.01 (6.67), 14.91–17.12
PS		
W0	2.33 (0.36), 2.28–2.38	2.25 (0.40), 2.19–2.30
W8	2.23 (0.37), 2.16–2.30	2.24 (0.40), 2.17–2.31
W16	2.09 (0.41), 2.00–2.17	2.17 (0.41), 2.09–2.25
W24	2.24 (0.35), 2.18–2.31	2.12 (0.41), 2.05–2.19
EQ-5D-3L (VAS)		
W0	73.17 (16.54), 70.91–75.42	72.42 (21.21), 69.40–75.45
W8	69.46 (20.48), 65.57–73.35	70.53 (23.46), 66.49–74.57
W16	70.26 (17.72), 66.44–74.08	72.61 (20.64), 68.74–76.47
W24	72.10 (19.70), 68.47–75.74	72.73 (23.28), 68.88–76.58

### 3.7 Primary outcomes

#### 3.7.1 Children’s social, emotional and behavioural difficulties

##### 3.7.1.1 Generalised linear model results

Group differences in SDQ scores over time were examined using generalised linear models. These analyses assessed the main effects of group (Embers vs. control), timepoint (baseline, 8, 16, and 24 weeks), and their interaction. Significant group effects were observed at 8 and 16 weeks, with the Embers condition reporting higher SDQ scores compared to the control condition. A significant interaction was observed at 24 weeks, indicating a greater reduction in SDQ scores over time in the Embers condition compared to the control condition. The full model results are presented in Table 3.

**Table 3:** Generalised linear model results for SDQ scores at 8, 16, and 24 weeks.

Timepoint comparison	Effect	*F*	*p*-value	95% CI (Lower, Upper)
Baseline vs. 8 Weeks	Overall model	*F* (3, 643) = 4.337	0.005	
	Group	*F* (1, 643) = 10.270	0.001	−2.943, −0.608
	Timepoint	*F* (1, 643) = 2.309	0.129	−0.404, 0.786
	Timepoint × Group	*F* (1, 643) = 0.360	0.549	−0.567, 1.066
Baseline vs. 16 Weeks	Overall Model	*F* (3, 599) = 3.023	0.029	
	Group	*F* (1, 599) = 6.357	0.012	−2.555, 0.144
	Timepoint	*F* (1, 599) = 0.070	0.792	−0.689, 0.884
	Timepoint × Group	*F* (1, 599) = 0.395	0.530	−1.388, 0.715
Baseline vs. 24 Weeks	Overall Model	*F* (3, 658) = 4.981	0.002	
	Group	*F* (1, 658) = 2.597	0.108	−1.553, 1.100
	Timepoint	*F* (1, 658) = 2.141	0.144	0.275, 1.770
	Timepoint × Group	*F* (1, 658) = 6.125	0.014	−2.306, −0.266

##### 3.7.1.2 SDQ within and between group contrasts (baseline vs. 24 weeks)

Within-group and between-group contrasts were conducted from baseline to 24 weeks. Participants in the Embers condition reported a significant reduction in their children’s emotional and behavioural difficulties, with SDQ scores decreasing from baseline (*M* = 17.21, *SD* = 4.02) to 24 weeks (*M* = 15.99, S*D* = 5.52), *t* (658) = 2.69, *p* = 0.007, 95% CI [0.275, 1.770], Cohen’s *d* = 0.10. In contrast, participants in the control condition did not report any improvement, with scores increasing slightly from baseline (*M* = 15.61, SD = 6.20) to 24 weeks (*M* = 16.01, SD = 6.67), *t* (658) = 0.75, *p* = 0.457, 95% CI [−0.957, 0.431], Cohen’s *d* = 0.03. Between-group comparisons revealed that at baseline, the Embers condition had significantly higher SDQ scores than the control, *t* (658) = 2.96, *p* = 0.003, contrast estimate = −1.512, 95% CI [−2.514, −0.510], Cohen’s *d* = 0.12. By 24 weeks, this difference was no longer statistically significant, *t* (658) = 0.34, *p* = 0.738, contrast estimate = −0.226, 95% CI [−1.553, 1.100], Cohen’s *d* = 0.01.

#### 3.7.2 Parental efficacy

##### 3.7.2.1 Generalised linear model results

Group differences in PSOC scores over time were examined using generalised linear models. Significant timepoint effects were observed at 8, 16, and 24 weeks, indicating improvements in PSOC scores across both groups. A significant timepoint × group interaction was observed at 16 weeks, with the Embers condition showing greater improvement compared to the control. No significant group effects were found at any timepoint. The full model results are presented in Table 4.

**Table 4:** Generalised linear model results for PSOC scores at 8, 16, and 24 weeks.

Timepoint comparison	Effect	*F*	*p*-value	95% CI (Lower, Upper)
Baseline vs. 8 Weeks	Overall model	*F* (3, 664) = 3.504	0.015	
	Group	*F* (1, 664) = 0.529	0.467	−0.650, 0.668
	Timepoint	*F* (1, 664) = 8.557	0.004	−1.130, −0.192
	Timepoint × Group	*F* (1, 664) = 1.320	0.251	−0.264, 1.010
Baseline vs. 16 Weeks	Overall model	*F* (3, 619) = 7.939	< 0.001	
	Group	*F* (1, 619) = 0.600	0.439	−1.564, −0.066
	Timepoint	*F* (1, 619) = 17.090	< 0.001	−2.004, −0.840
	Timepoint × Group	*F* (1, 619) = 9.206	0.003	0.425, 1.983
Baseline vs. 24 Weeks	Overall model	*F* (3, 676) = 8.998	< 0.001	
	Group	*F* (1, 676) = 0.003	0.956	−1.011, 0.401
	Timepoint	*F* (1, 676) = 24.341	< 0.001	−1.695, −0.683
	Timepoint × Group	*F* (1, 676) = 3.300	0.070	−0.052, 1.333

##### 3.7.2.2 PSOC within and between group contrasts (baseline vs. 16 weeks)

Within-group and between-group contrasts were conducted from baseline to 16 weeks. Participants in the Embers condition reported a significant improvement in parenting sense of efficacy, with PSOC scores increasing from baseline (*M* = 19.30, SD = 3.16) to 16 weeks (*M* = 20.64, SD = 2.73), *t* (619) = 4.80, *p* < 0.001, 95% CI [−2.004, −0.840], Cohen’s *d* = 0.26. In contrast, participants in the control condition did not report any significant change, with scores remaining relatively stable from baseline (*M* = 19.71, SD = 2.76) to 16 weeks (*M* = 19.92, SD = 2.95), *t* (619) = 0.83, *p* = 0.409, 95% CI [−0.736, 0.300], Cohen’s *d* = 0.03. Between-group comparisons revealed that at baseline, the Embers condition had slightly lower PSOC scores than the control, although this difference was not statistically significant, *t* (619) = 1.34, *p* = 0.181, contrast estimate = 0.389, 95% CI [−0.181, 0.959], Cohen’s *d* = 0.08. By 16 weeks, the Embers condition had significantly higher PSOC scores than the control, *t* (619) = 2.14, *p* = 0.033, contrast estimate = −0.815, 95% CI [−1.564, −0.066], Cohen’s *d* = 0.12.

##### 3.7.2.3 PSOC within and between group contrasts (baseline vs. 24 weeks)

Within-group and between-group contrasts were conducted from baseline to 24 weeks. Participants in the Embers condition reported a significant improvement in parenting sense of efficacy, with PSOC scores increasing from baseline (*M* = 19.30, SD = 3.16) to 24 weeks (*M* = 20.45, SD = 3.25), *t* (676) = 4.61, *p* < 0.001, 95% CI [−1.695, −0.683], Cohen’s *d* = 0.18. The control condition also showed a significant increase in scores from baseline (*M* = 19.71, SD = 2.76) to 24 weeks (*M* = 20.24, SD = 2.95), *t* (676) = 2.28, *p* = 0.023, 95% CI [−1.020, −0.077], Cohen’s *d* = 0.09. Between-group comparisons revealed no significant differences at baseline, *t* (676) = 1.16, *p* = 0.246, contrast estimate = 0.336, 95% CI [−0.232, 0.903], Cohen’s *d* = 0.04, or at 24 weeks, *t* (676) = −0.85, *p* = 0.396, contrast estimate = −0.305, 95% CI [−1.011, 0.401], Cohen’s *d* = 0.03.

#### 3.7.3 Parental discipline

##### 3.7.3.1 Generalised linear model results

Group differences in Parenting Scale (PS) scores over time were examined using generalised linear models. These analyses assessed the main effects of group (Embers vs. control), timepoint (baseline, 8, 16, and 24 weeks), and their interaction. Significant timepoint effects were observed at 8 weeks, *F* (1, 654) = 9.33, *p* = 0.002; 16 weeks, *F* (1, 607) = 39.62, *p* < 0.001; and 24 weeks, *F* (1, 666) = 23.00, *p* < 0.001, indicating reductions in PS scores over time across both groups. A significant timepoint × group interaction was observed at 8 weeks, *F* (1, 654) = 5.01, *p* = 0.026, and at 16 weeks, *F* (1, 607) = 10.98, *p* < 0.001, suggesting greater reductions in the Embers condition. A significant group effect was observed at 24 weeks, *F* (1, 666) = 8.59, *p* = 0.003, with the Embers condition reporting lower PS scores compared to the control. The full model results are presented in Table 5.

**Table 5:** Generalised linear model results for PS scores at 8, 16, and 24 weeks.

Timepoint comparison	Effect	F	*p*-value	95% CI (Lower, Upper)
Baseline vs. 8 Weeks	Overall model	*F* (3, 654) = 5.162	0.002	
	Group	*F* (1, 654) = 0.709	0.400	−0.077, 0.099
	Timepoint	*F* (1, 654) = 9.325	0.002	0.045, 0.155
	Timepoint × Group	*F* (1, 654) = 5.005	0.026	−0.159, −0.010
Baseline vs. 16 Weeks	Overall model	*F* (3, 607) = 16.180	< 0.001	
	Group	*F* (1, 607) = 0.005	0.941	−0.029, 0.169
	Timepoint	*F* (1, 607) = 39.619	< 0.001	0.146, 0.275
	Timepoint × Group	*F* (1, 607) = 10.976	< 0.001	−0.232, −0.059
Baseline vs. 24 Weeks	Overall Model	*F* (3, 666) = 11.769	< 0.001	
	Group	*F* (1, 666) = 8.593	0.003	−0.226, −0.051
	Timepoint	*F* (1, 666) = 23.002	< 0.001	0.005, 0.127
	Timepoint × Group	*F* (1, 666) = 2.825	0.093	−0.012, 0.154

##### 3.7.3.2 PS within and between group contrasts (baseline vs. 16 weeks)

Within-group and between-group contrasts were conducted from baseline to 16 weeks. Participants in the Embers condition reported a significant reduction in negative parental discipline styles, with PS scores decreasing from baseline to 16 weeks, *t* (607) = 6.42, *p* < 0.001, contrast estimate = 0.211, 95% CI [0.146, 0.275], Cohen’s *d* = 0.26. The control condition also showed a significant reduction in PS scores from baseline to 16 weeks, *t* (607) = 2.25, *p* = 0.025, contrast estimate = 0.065, 95% CI [0.008, 0.123], Cohen’s *d* = 0.09. Between-group comparisons revealed that at baseline, the Embers condition had significantly lower PS scores than the control, *t* (607) = 2.02, *p* = 0.044, contrast estimate = −0.076, 95% CI [−0.149, −0.002], Cohen’s *d = 1.45.* By 16 weeks, this difference was no longer statistically significant, *t* (607) = 1.39, *p* = 0.165, contrast estimate = 0.070, 95% CI [−0.029, 0.169], Cohen’s *d* = 0.99.

##### 3.7.3.3 PS within and between group contrasts (baseline vs. 24 weeks)

Within-group and between-group contrasts were conducted from baseline to 24 weeks. Participants in the Embers condition reported a significant reduction in negative parental discipline styles, with PS scores decreasing from baseline to 24 weeks, *t*(666) = 2.13, *p* = 0.034, contrast estimate = 0.066, 95% CI [−0.005, 0.127], Cohen’s *d* = 0.08. The control condition also showed a significant reduction in PS scores from baseline to 24 weeks, *t* (666) = 4.75, *p* < 0.001, contrast estimate = 0.137, 95% CI [0.080, 0.194], Cohen’s *d* = 0.18. Between-group comparisons revealed no significant difference at baseline, *t* (666) = 1.81, *p* = 0.071, contrast estimate = −0.067, 95% CI [−0.141, 0.006], Cohen’s *d* = 1.81. However, by 24 weeks, the Embers condition had significantly lower PS scores than the control, *t* (666) = 3.12, *p* = 0.002, contrast estimate = −0.139, 95% CI [−0.226, −0.051], Cohen’s *d* = 0.32.

#### 3.7.4 EQ-5D-3L (vas)

##### 3.7.4.1 Generalised linear model results

Group differences in EQ-5D-3L Visual Analogue Scale (VAS) scores over time were examined using generalised linear models. These analyses assessed the main effects of condition (Embers vs. control), timepoint (baseline, 8, 16, and 24 weeks), and their interaction. A significant main effect of timepoint was observed at 8 weeks, *F* (1, 637) = 4.03, *p* = 0.045, indicating a change in VAS scores over time. However, no significant timepoint effects were found at 16 or 24 weeks, and no significant group effects or timepoint × group interactions were observed at any timepoint (all *p*s > 0.10). Within-group and between-group contrasts from baseline to 16 and 24 weeks were also non-significant, suggesting that VAS scores remained stable over time in both conditions. The full model results are presented in Table 6.

**Table 6:** Generalised linear model results for EQ-5D-3L (VAS) scores at 8, 16, and 24 weeks.

Timepoint comparison	Effect	*F*	*p*-value	95% CI (Lower, Upper)
Baseline vs. 8 Weeks	Overall Model	*F* (3, 637) = 1.484	0.218	
	Group	*F* (1, 637) = 0.001	0.975	−4.324, 6.296
	Timepoint	*F* (1, 637) = 4.032	0.045	−0.230, 7.481
	Timepoint × Group	*F* (1, 637) = 0.474	0.491	−7.132, 3.429
Baseline vs. 16 Weeks	Overall Model	*F* (3, 593) = 0.970	0.407	
	Group	*F* (1, 593) = 0.484	0.487	−1.677, 8.589
	Timepoint	*F* (1, 593) = 0.500	0.480	−0.807, 7.086
	Timepoint × Group	*F* (1, 593) = 2.583	0.109	−9.688, 0.968
Baseline vs. 24 Weeks	Overall Model	*F* (3, 654) = 0.227	0.877	
	Group	*F* (1, 654) = 0.001	0.978	−4.303, 6.049
	Timepoint	*F* (1, 654) = 0.182	0.670	−2.436, 5.458
	Timepoint × Group	*F* (1, 654) = 0.452	0.502	−7.249, 3.552

### 3.8 Health economics analysis

Data from the participant survey were collected to estimate efficacy of Embers compared to TAU resource use, including the direct costs associated with; utilisation GP appointments, contact with social services and contact with family liaison services. Additionally, out of pocket household related direct costs associated with healthcare expenditure were also collected. To calculate the total costs per trial arm, unit costs derived from the Unit Costs of Health and Social Care (Jones et al., 2024) were assigned to the number of GP appointments, contact with social services and contact with family liaison services, with the cost of Embers being estimated from Embers internal data (Table 7).

**Table 7:** Outcome data model inputs.

Effects	Embers	Treatment as usual	Source
Change in SDQ	−1.25	0.41	Current study
Change in PSOC	1	0.53	
Utility	0.88	0.85	
GP appointment utilisation	0.16	0.19	
Contact with social services	0.02	0.05	
Contact with family liaison services	0.2	0.46	

A decision tree model was developed to estimate the costs and effects of Embers compared to TAU for parents enrolled in the Embers trial. This model was used to estimate the cost-consequence of Embers, namely the cost per point improvement in PSOC and the SDQ scores. 1,000 model simulations were run to estimate uncertainty in the model (Table 8).

**Table 8:** Cost data model inputs.

Costs	Embers (£)	Treatment as usual (£)	Source
Cost of intervention	2.70	0	Embers internal data
GP appointment	45	45	PSSRU (Jones et al., 2024)
Contact with social services	106	106	(Goodman *et al.,* 2017)
Contact with family liaison services	106	106	Assumed equal to social services
Out of pocket expenditure	271.57	392.49	Current study

Probabilistic sensitivity analyses were conducted alongside the production of cost-effectiveness acceptability curves, which demonstrated that the Embers intervention had a 98% probability of being cost-effective at conventional willingness-to-pay thresholds (Table 9).

**Table 9:** Health economics outcomes.

	Embers [mean (95% CI)]	Treatment as usual [mean (95% CI)]
Total cost (£)	303 (199, 430)	459 (325, 624)
Δ PSOC	1.01 (0.602, 1.38)	0.523 (0.132, 0.928)
ΔSDQ		
Utility	0.88 (0.781, 0.979)	0.849 (0.749, 0.947)
ICER (cost per 1 point improvement in PSOC)	−293.56	
ICER (cost per 1 point improvement in SDQ)	−85.70	

Embers is less costly and more effective than treatment as usual. At a willingness to pay of £200 per point improvement in PSOC, Embers is 98% likely to be cost-effective. The cost of 1 point improvement in SDQ was estimated at -£86, which indicates that Embers generates cost-savings whilst improving outcomes. The cost of 1 point improvement in PSOC was estimated at -£294 It is therefore less expensive to provide Embers than treatment as usual, while improving outcomes for parents. Although the SDQ shows a much larger improvement than PSOC, the ICER appears smaller because the same cost savings are spread over a bigger effect, resulting in lower savings per unit of improvement.

The mean ICER of the 1,000 probabilistic simulations is -£86, which represents the cost per 1 point improvement in SDQ.

**Figure 2:**
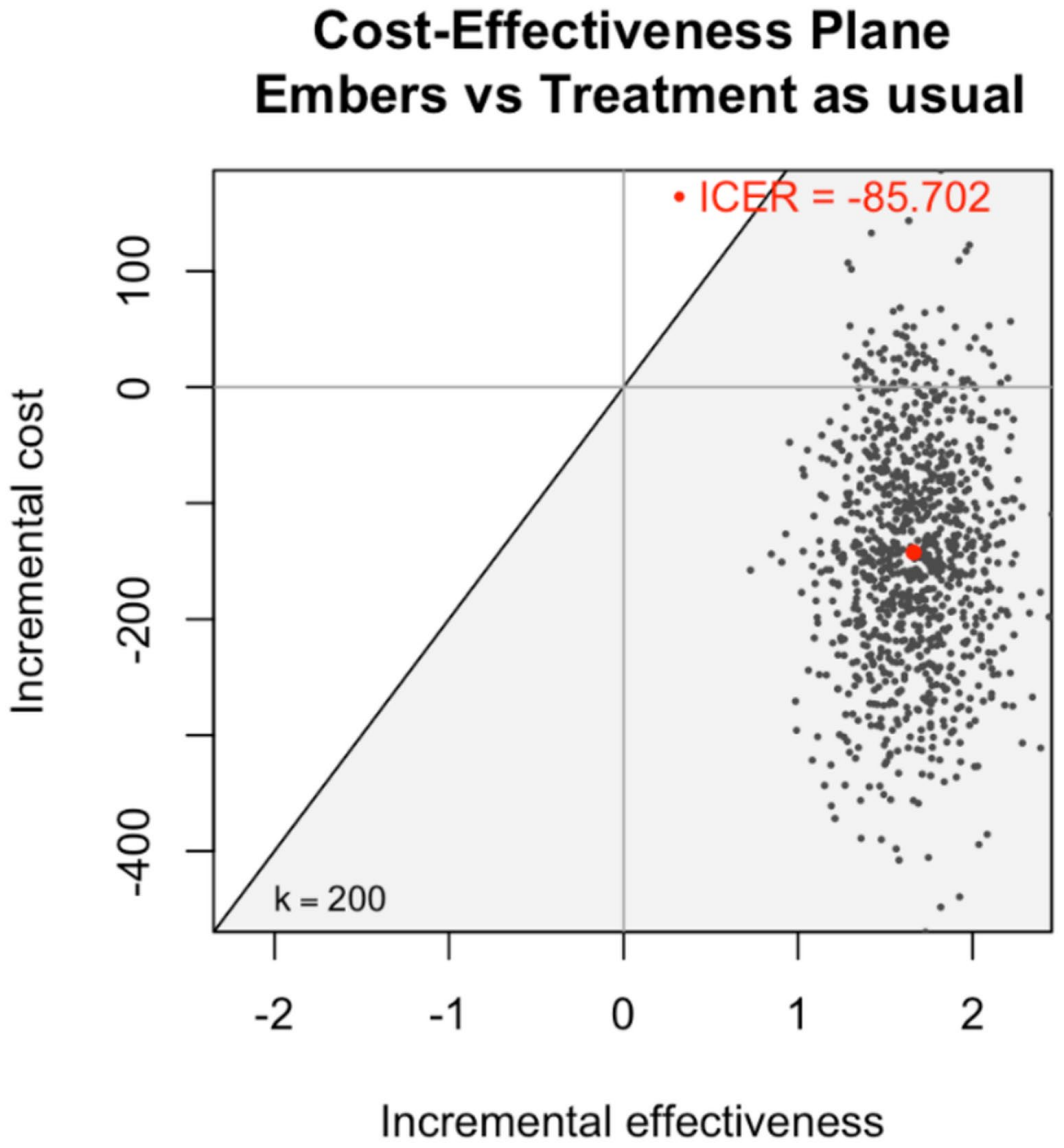
Cost-effectiveness plane, cost per 1 point improvement in SDQ.

**Figure 3:**
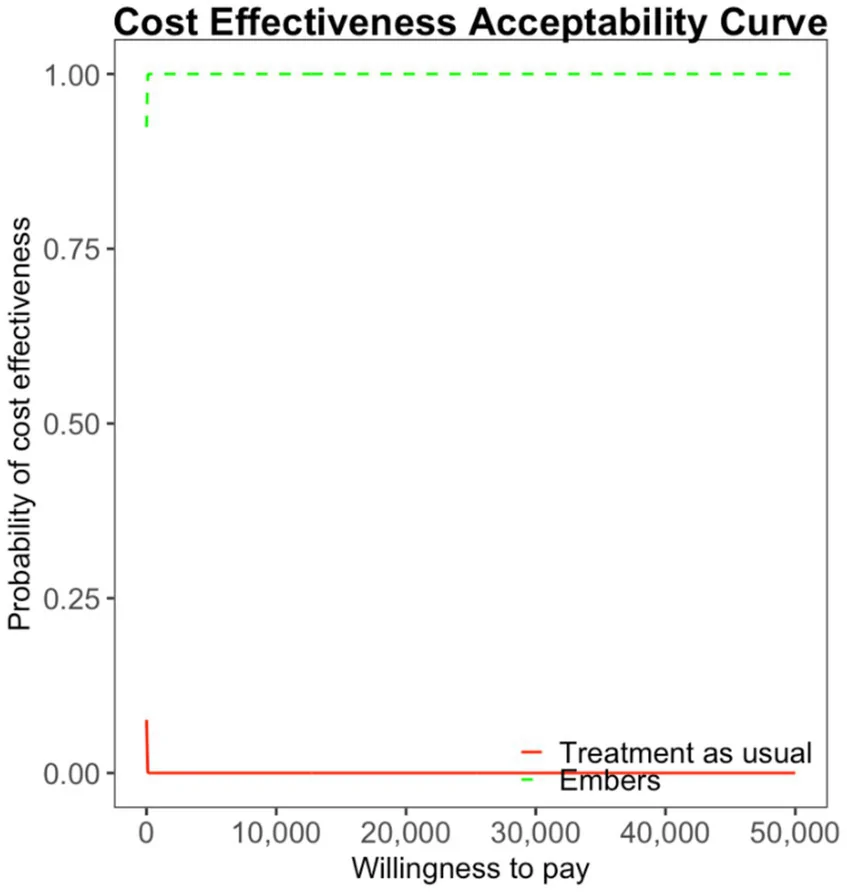
Cost-effectiveness acceptability curve, cost per 1 point improvement in SDQ.

**Figure 4:**
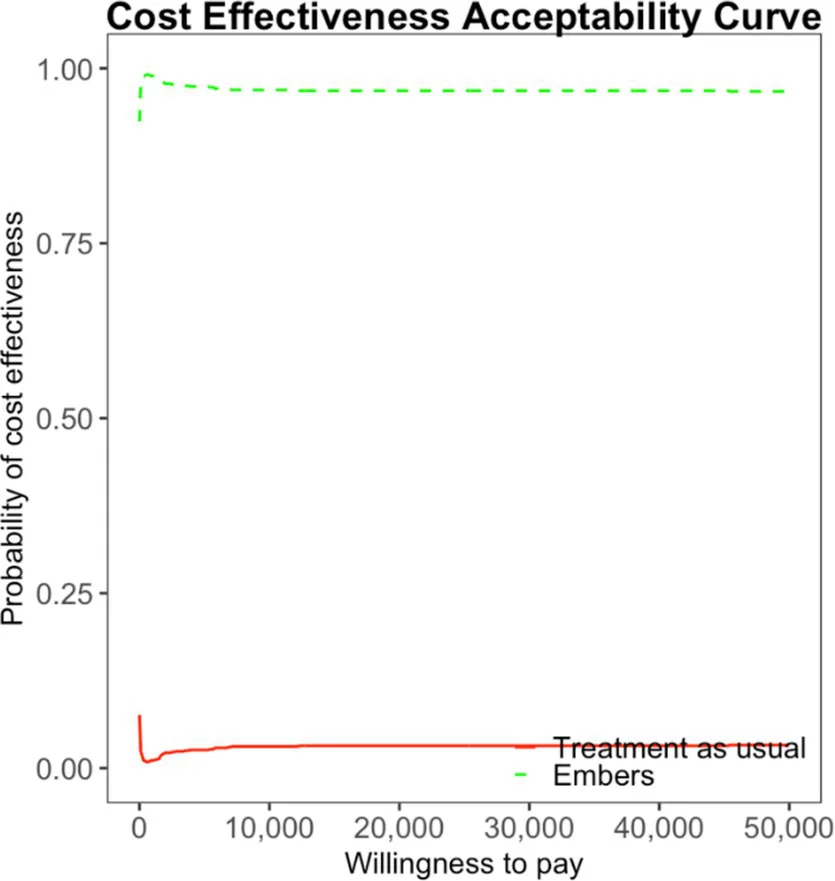
Cost-effectiveness acceptability curve, cost per 1 point improvement in PSOC.

**Figure 5:**
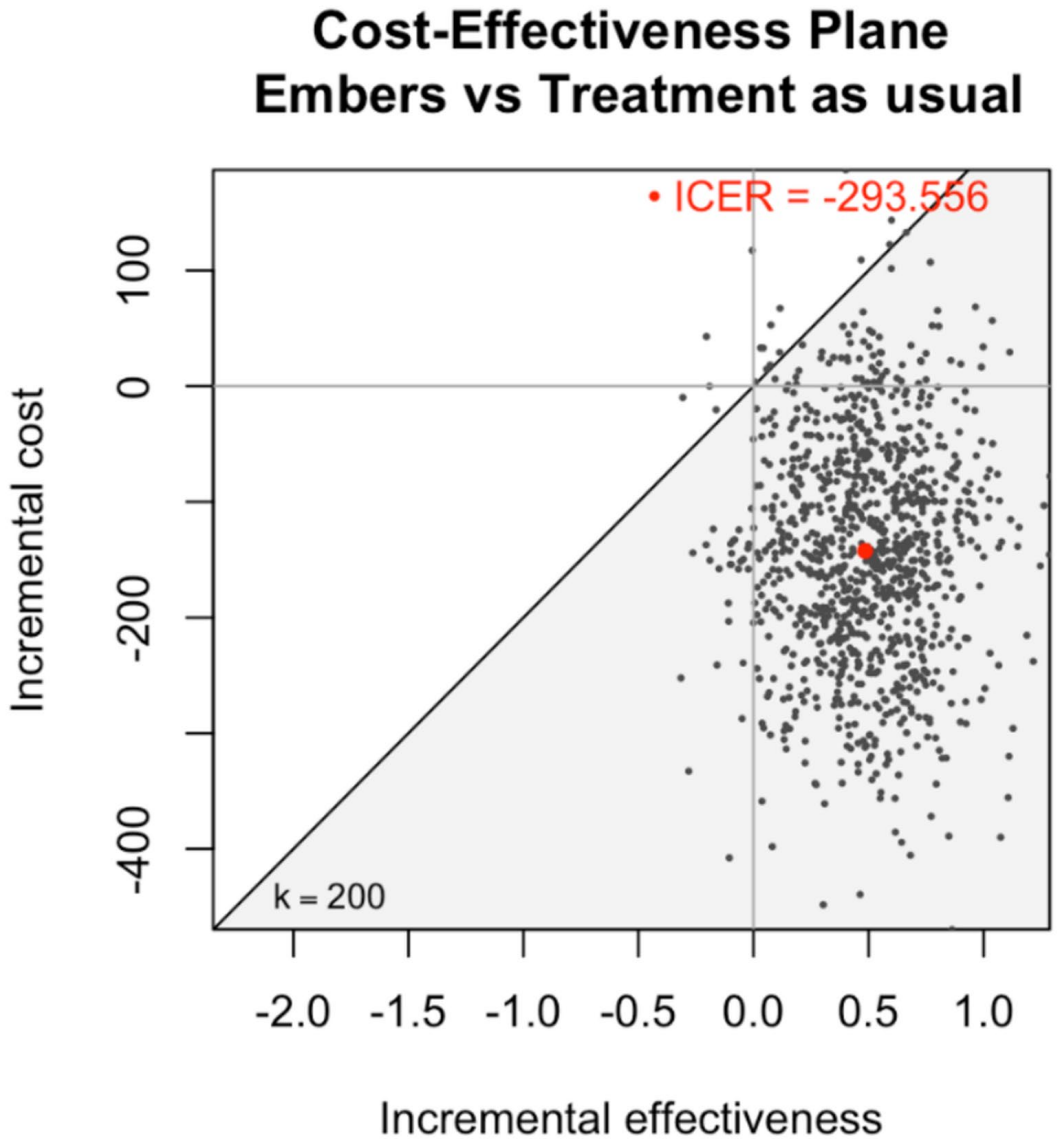
Cost-effectiveness plane, cost per 1 point improvement in PSOC.

The mean ICER of the 1,000 probabilistic simulations is -£294, which represents the cost per 1 point improvement in PSOC (Figures 2–5).

## 4 Discussion

### 4.1 Primary findings

This current study evaluated the efficacy and health economic benefit of Embers the Dragon, a self-guided digital program designed to support the development of children aged 4–7 and the parenting confidence of their parents/guardians. Participants in the Embers condition demonstrated statistically significant improvements in terms of children’s development shown by a decrease in difficulties, an increase in parenting confidence and reductions in negative parenting discipline style. This gain was significantly greater than participants in the baseline (treatment as usual) condition for one of the primary outcomes, children’s development. The findings contribute to a growing body of literature supporting the use of digital mental health interventions (DMHI)s to address the increasing demand for early mental health support in young children, particularly in the context of limited access to traditional services.

The Embers condition showed a reduction in child emotional and behavioural difficulties, with SDQ scores decreasing between baseline and 24 weeks. Although the effect size was modest, it was clinically meaningful, reflecting a shift from the ‘high’ clinical range (≥17) to the ‘slightly raised’ range (14–16), as defined by UK SDQ scoring guidelines (Suffolk County Council, 2024). This threshold shift is significant, as scores of 17 or above indicate a cause for concern’, prompting consideration of additional support or referral, potentially involving multi-agency discussions and services like CAMHS (London Borough of Bexley, ONFTSELICS, 2023; Suffolk County Council, 2024). In contrast, scores in the ‘slightly raised’ range may warrant monitoring, but not necessarily referral or intervention. Reducing the number of children in the high-risk category may help alleviate pressure on overstretched services, improve access for those with more acute needs, and enable earlier, lower-intensity support. The effect size observed in our trial is consistent with those reported in similar digital mental health intervention (DMHI) studies using the SDQ as an outcome measure. For instance, a recent systematic review of randomised controlled trials found that SDQ-based effect sizes typically range from 0.10 to 0.30, which are small to modest improvements that, importantly, can translate into meaningful clinical change (Saboor et al., 2024). These findings support the validity of our interpretation of the observed effects and suggest that the Embers intervention performs comparably within the broader landscape of digital approaches targeting child and adolescent mental health. For this outcome, these gains were larger in the Embers condition than in the control condition.

Improved parental confidence was also observed, with the Embers condition demonstrating a statistically significant improvement in PSOC scores between baseline and 24 weeks. While this effect size is smaller than those reported in other similar parenting interventions (Canário et al., 2025; Piotrowska et al., 2020), it does reflect meaningful gains in parenting confidence, particularly in the context of a scalable, low-intensity approach. It is also important to note that, although improvements in Embers were significantly greater than control at 16 weeks, by 24 weeks, participants in the control condition also showed a significant improvement in PSOC scores, and the interaction term and between-group differences were no longer statistically significant. This suggests that factors beyond the intervention itself, such as increased self-awareness from completing parenting questionnaires, the influence of study participation, or natural developmental changes in children, may have contributed to improvements in parenting confidence across both groups. It also requires caution to be taken in interpreting the effect of the intervention on confidence. This effect may also have been influenced by the additional reminders at 24 weeks (increasing participation rates). If this is the case, it could be that reduced engagement over time played a role in the varying impact of the intervention on confidence (noting the significant difference in SDQ scores remain).

Similarly, reductions in negative parental discipline behaviours were observed in both conditions at 8, 16, and 24 weeks, suggesting that participation in the study and other factors beyond the intervention itself may have influenced the findings. However, the Embers condition saw increased reduction relative to control at 8 and 16 weeks, had consistently more pronounced reductions in PS scores across time, and at 24 weeks scores in this condition were significantly lower compared to their control counterparts with a small but meaningful effect size (Cohen’s *d* = 0.08) - although the interaction term became marginal. Although not definitive, these results highlight the possibility that the intervention may be effective in alleviating negative parental discipline styles, a key factor in supporting child wellbeing and family functioning.

Finally, no meaningful changes were observed in parental quality of life as measured by the EQ-5D-3L. This finding was not unexpected, given that the measure is designed to assess general health-related quality of life and does not specifically target domains relevant to parenting or the intervention itself. The measure was included to support health economic modelling, rather than to capture direct outcomes of the intervention.

It is important to consider how the current findings contribute to the existing evidence base. Embers was found to be associated with improvements in child emotional and behavioural difficulties, alongside positive changes in parenting, which are broadly comparable to those reported in well-established parenting interventions such as ‘Triple P’ and ‘Family Check-Up’. These findings are particularly encouraging given the low-intensity format of the Embers intervention. Embers is delivered as a fully online, self-guided programme, and this flexible and accessible approach represents a scalable model that can achieve clinically meaningful improvements while also helping to reduce common barriers to engaging with more resource-intensive or practitioner-led support. By supporting improvements in child emotional and behavioural difficulties, as well as parenting outcomes, at an earlier stage and at lower intensity, this intervention format may help to reduce pressure on already overstretched services (e.g., CAMHS) by limiting the number of children requiring more intensive support, while also improving access to support for families who may face challenges in accessing care.

Beyond this, Embers incorporates both parent and child components, supporting change across the family system rather than focusing solely on parenting behaviour. This is a notable feature, as relatively few digital mental health interventions include both parent and child components within a single programme (Chew et al., 2026). This reflects the gap identified in the current literature and suggests that integrating both components within a scalable digital format may be a useful direction for future intervention development. Targeting multiple aspects of the family system in this way may strengthen the pathways through which improvements in child outcomes are achieved and maintained over time.

Our health economic analysis estimated the cost effectiveness of Embers compared to TAU, which found that Embers is both less costly and more effective in improving SDQ and PSOC scores than TAU. This was driven by a reduction in resource utilisation and out of pocket costs compared to TAU, which highlights societal and household cost savings, freeing up health and social care resources whilst benefitting individual households economically. However, it was not possible to estimate the full range of costs and outcomes due to the limited available data. Furthermore, only a one-year time horizon could be considered due to the lack of long-term cost and outcome data. Future analysis should focus on long-term costs and outcomes for parents, and a wider range of costs and outcomes to fully estimate the cost-effectiveness of Embers compared to TAU, such as use of mental health services, using a more robust model structure such as a Markov Cohort model to estimate the cost-effectiveness of Embers over the lifetime horizon.

### 4.2 Limitations

This study has a few limitations. Attrition was higher in the Embers group (44.7%) than in controls (32.7%). While missing data were accounted for via multilevel modelling (including the possibility of data missing not at random), differential dropout across participants may have introduced bias. High attrition is common in DMHI trials, particularly app-based interventions, and reflects challenges such as low interactivity and competing demands (Boucher and Raiker, 2024; Prior et al., 2023; Smith et al., 2025). It may also be the case that the improvements observed in the Embers condition were themselves a driver of lower engagement (as the need for the intervention had been met). Despite PPI informed materials, sustaining engagement over 24 weeks remained challenging, suggesting future work should explore gamification, adaptive content, or dynamic strategies.

Second, in common with other research in this area (Di Pierdomenico et al., 2025) all outcomes were parent reported. While appropriate given the age of participants (4–7 years), this approach captures parental perceptions rather than objective indicators of change. Children’s perspectives were collected separately through qualitative interviews (alongside parents). Importantly, children’s perspectives were captured in the current study through qualitative interviews, and these findings will be reported in a separate paper (which will also be lodged on the project OSF site).

It should be noted we used a modified version of the SDQ. This was due to the 6-month change period specified in standard scale not being sufficiently sensitive to detect changes in our study timeframes (see also *Footnote 1*, above). This is a limitation as it precludes direct comparability with studies that rely solely on the unmodified six-month SDQ. However, it does aligns with other research that uses the one-month version at follow-up assessments (e.g., Cooper et al., 2010; Joyce et al., 2023).

This point also relates to a limitation around the study follow-up period, which was 24 weeks. Longer term sustaining of impacts of the intervention cannot be assumed given the current data.

Finally, the sample was predominantly white, heterosexual, married mothers, limiting generalisability. Underrepresentation of minority and disadvantaged groups is a persistent issue in mental health research (Buchanan and Holly, 2025; Jukes et al., 2024) despite their disproportionate need (Bains and Gutman, 2021; Collins Nwannebuike Nwokedi et al., 2025; Ekezie et al., 2024; Redwood and Gill, 2013). To address this, recruitment was conducted nationally, with postcode data used to derive deprivation indices for health economics analysis. Community organisations were approached but engagement was limited. Inclusive materials were developed with PPI input, though further refinement and codesign may improve reach. Variance in deprivation was also controlled for in the analysis. Future work should prioritise inclusive recruitment, culturally sensitive outreach, flexible participation formats, and systematic monitoring of diversity metrics to examine how factors such as ethnicity, socioeconomic status, and digital literacy influence engagement. Whether responses to DMHIs are dose-dependent, and which elements are driving positive effect also remains an open question. Such and analysis was not possible as an exploratory analysis in the current study due to the range of metrics recorded – it also noting variance in usage was accounted for in the model.

While not a limitation, it should be noted that the current project had a commercial partner (see Section 8; Funding) and the nature of the project required initial input from them in terms of study design. The delivery of trial, statistical analysis planning and implementation, interpretation of findings and preparation were independent. A number of checks, balances and controls were put in place to ensure study independence – including a trial steering panel, protocol publication, OSF pre-registration (of the design and study analysis plans) and ensuring LSBU were contractually free to publish the findings regardless of outcome.

### 4.3 Implications

As well as clear evidence that the Embers platform is beneficial for children and parents, the observed improvements in child emotional and behavioural outcomes, alongside reductions in negative parental discipline styles, provide encouraging evidence for the wider potential of low-intensity, self-guided DMHIs to support children experiencing mild to moderate mental health concerns. This study also adds to the limited but growing evidence base for dual-focused digital interventions that engage both parents and children. Collaborative models of this kind may enhance engagement, reinforce learning, and support the integration of strategies into everyday routines. Both the cost-effectiveness and potential for integration into targeted treatment pathways and support services makes interventions like Embers the Dragon particularly relevant within public health and education systems. As the platform is referral agnostic, it may be particularly useful in for multi-agency/disciplinary contexts, such as neighbourhood care teams. Due to persistent challenges in accessing traditional services, including long waiting times and limited capacity, accessible digital tools could serve as preventative supports or be embedded as adjuncts to existing care pathways, helping to reduce pressure on overstretched systems and reach families earlier in the care pathway.

The trial also highlights key implementation challenges, including participant attrition and low school engagement. Only 0.5% of UK schools contacted agreed to participate, which prevented comparison of school-based versus home-based delivery. This highlights barriers to embedding interventions in education settings, and limits generalisability of the current efficacy findings to settings beyond home-based delivery, as well as suggesting the importance of parent motivation. Future research should revisit school-based implementation to assess scalability and the added value of delivery in schools compared to home use. The low uptake of schools may reflect resource constraints (e.g., time), which schools often cited as a barrier, even when they expressed interest in participating.

These findings underscore the need for more dynamic engagement strategies and alternative delivery models, such as hybrid formats or frictionless school-based integration. Future research should explore long-term outcomes, mechanisms of change, and differential effects across diverse populations to optimise impact and ensure equitable access.

## 5 Conclusion

The current trial found that the intervention delivered clinically meaningful benefits to children and their families through a low-intensity, self-guided digital format, outperforming control participants in terms of children’s developmental wellbeing. The findings align with broader trends in DMHI research and specifically highlight the potential of dual-focused digital tools to complement existing treatment pathways (for which limited evidence exists). The scalable design and accessibility of Embers the Dragon make it a viable option for supporting early childhood mental health, particularly in settings where access to traditional services is limited. Further research is needed to enhance impact, improve engagement, and ensure the intervention reaches families who may benefit most.

## Data Availability

The datasets presented in this study can be found in online repositories. The names of the repository/repositories and accession number(s) can be found at: https://osf.io/4xjt9.

## Footnotes

1
https://osf.io/ybxzu/

2 see pre-registration, https://osf.io/e5c9g.

3 OSF; https://osf.io/ybxzu/.

4 https://osf.io/xzaj7/.

5 Modifying the SDQ is not permitted under the SDQ terms of use (see Copyright Notice on https://www.sdqinfo.org/) and means the results are not directly comparable with other studies using the SDQ. The authors regret this error and commit to abide fully by the SDQ terms of use in future collection of SDQ data.
