# Correction: An online randomised controlled trial to evaluate the effectiveness of an online intervention for parents/guardians of children aged 4–7 years old who are concerned about their children's emotional and behavioural development: EMERGENT study

**DOI:** 10.3389/fpsyg.2026.2023306

**Published:** 2026-10-28

**Authors:** Jowinn Chew, Daniel Frings, Paula Reavey, Clare Allabyrne, Claudiu Herteliu, Chris Flood, Lucy Watson

**Affiliations:** 1 London South Bank University, London, United Kingdom; 2 Bucharest University of Economic Studies, Bucharest, Romania

**Keywords:** children, digital health, digital mental health interventions, mental well-being, parental intervention, randomised controlled trials

A *F* value was reported incorrectly in the original submission. In **Table 3**, the *F* value for the Baseline vs. 8 week interaction was reported as 4.337 (**Table 3**, Row 3). The correct value is *F* = 0.360. The *p* values, and 95% confidence intervals are correct, and the change does not impact patterns of significance nor interpretation.

The original version of this article has been updated.

